# Computed tomography angiography assessment of the degree of simple coarctation of the aorta and its relationship with surgical outcome: A retrospective analysis

**DOI:** 10.3389/fped.2022.1017455

**Published:** 2022-12-05

**Authors:** Hui-Jun Xiao, A-Lai Zhan, Qing-Wen Huang, Rui-Gang Huang, Wei-Hua Lin

**Affiliations:** Department of Radiology, Zhangzhou Affiliated Hospital of Fujian Medical University, Zhangzhou, China

**Keywords:** computed tomography angiography, coarctation of the aorta, surgical outcome, infant, prognosis

## Abstract

**Objective:**

To investigate the correlation between the degree of aortic coarctation and surgical prognosis in infants with simple coarctation of the aorta (CoA) using computed tomography angiography (CTA).

**Methods:**

This study was a retrospective study. Twenty-seven infants with simple CoA who underwent surgical correction from January 2020 to June 2022 were enrolled. Aortic diameters were measured at five different levels and normalized to Z scores based on the square root of body surface area. The relevant data were collected and analyzed, and the predictors associated with surgical outcome were determined.

**Results:**

Patients were divided into the mild CoA group and the severe CoA group according to the severity of coarctation. The mechanical ventilation duration and the length of ICU stay in the mild CoA group were significantly lower than those in the severe CoA group. Multiple linear regression analyses revealed that the degree of aortic coarctation was a significant risk factor for a prolonged postoperative ICU stay. In addition, gestational age and age at operation were risk factors for a prolonged postoperative ICU stay. Correlation analysis showed that the degree of aortic coarctation correlated with the Z scores of the ascending aorta and postcoarctation aorta.

**Conclusion:**

The degree of the CoA is an important predictor of surgical outcomes in infants with simple CoA and was significantly correlated with the ascending aorta and postcoarctation aorta Z scores. Therefore, preoperative CTA should be routinely performed to assess the degree of aortic coarctation and better identify risk factors.

## Introduction

Congenital heart disease (CHD) is one of the most common congenital malformations, and coarctation of the aorta (CoA) accounts for approximately 5%–10% of live births with CHD (1, 2). CoA can be simple or complex with other cardiovascular malformations, such as patent ductus arteriosus, atrial septal defect, ventricular septal defect, transposition of the great arteries, etc (3, 4). Surgical correction is currently generally recommended in early infancy for optimal treatment outcomes. Currently, patients with simple CoA mainly undergo surgical correction safely and effectively through the lateral thoracotomy approach. Crafoord first performed 4 surgical treatments of CoA in 1944 (5). Numerous studies have assessed the prognosis of infants with CoA. Kumar et al. showed that the early blood pressure gradient after aortic surgery was a significant predictor of recoarctation in the long term (6). Schoeneberg's study on the influencing factors of postoperative hospital stay in children with CoA showed that preterm birth (before 37 weeks of gestation) was an independent risk factor for postoperative hospital stay and postoperative mortality (7). However, a literature search found few relevant studies on the degree of aortic coarctation on early prognosis after surgical correction in infants. Therefore, we conducted this retrospective study to determine the reliability of the degree of aortic coarctation as a predictor of surgical outcome and to identify further factors associated with the outcome of surgical correction of simple CoA.

## Methods

This study was a retrospective study of 27 infant patients who underwent surgical correction of simple CoA between January 2020 and June 2022. In all these infants, the final diagnosis was made with reference to preoperative transthoracic echocardiography (TTE) and computed tomography angiography (CTA) findings. In CTA images, the coarctation site-diaphragm ratio (CDR) was used to describe the degree of aortic coarctation (8). According to the severity of the coarctation, all the patients were divided into a mild CoA group (CDR > 50%, 13 cases) and a severe CoA group (CDR < 50%, 14 cases). The institutional ethics committee of our hospital approved our study. Inclusion criteria: 1. the patients were younger than one year old; 2. infants who underwent surgical correction of simple CoA; 3. all patients underwent TTE and CTA examination before the operation. Exclusion criteria: 1. cases with incomplete clinical data; 2. complex CoA with other intracardiac or extracardiac malformations; 3. patients complicated with other systemic diseases.

## Computed tomography angiography

All the infant patients in this study underwent CTA examination using GE Revolution CT with 256 rows. Scanned image postprocessing was performed using the GE SERVICE AW4.7 intelligent advanced postprocessing workstation. CT aorta scanning protocol: Infant patients were scanned in a sleeping state with calm breathing. The patients who could not cooperate were fasted for 4–6 h before the examination and were orally sedated with 10% chloral hydrate (0.5 ml/kg) 30–60 min before the examination and then scanned after they fell into a deep sleep. Indwelling needles were placed in the antecubital or dorsal hand vein in all patients. The patient was placed in the supine position, and the scanning direction was from head to foot, ranging from the thoracic entrance to the level of the pubic symphysis. The layer thickness of the scanned image was 0.625 mm, and the layer spacing was 0.625 mm. Scanning parameters: tube voltage and current were adjusted according to the patient's body mass index. Tube voltage was 80–100 kV, and tube current was 80–120 mas. The nonionic isotonic agent iodixanol (320 mg/ml) was used, and the injection dose was calculated according to the body weight of 1.5–2 ml/kg. Contrast media was injected through the indwelling needle at a rate of 1.0–2.0 ml/s (according to the patient's age, height, and weight), and then 10–15 ml of normal saline was injected at the same rate. To monitor the CT value, the contrast-tracer method was used to select the region of interest (ROI) at the tracheal carina of the descending aorta. When the CT value in the ROI reached 90–100 HU, the scan was automatically triggered after a 5-second delay. The pitch was adjusted according to the heart rate. The scanning layer thickness was 0.625 mm, and the scanning interval was 0.312 mm.

**Figure 1:**
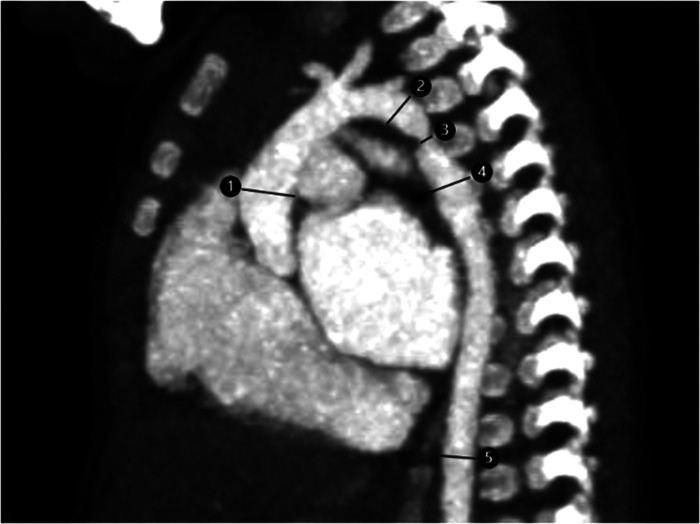
Ct image of aortic coarctation in a 2-month-old infant. 1. ascending aorta; 2. pre-coarctation aorta; 3. site of coarctation; 4. post-coarctation aorta; 5. descending aorta at level of diaphragm.

The scanned images were processed in the image postprocessing workstation, and 2D and 3D images were postprocessed and reconstructed. The postprocessing reconstruction included multiple planar reconstruction (MPR), maximum intensity projection (MIP), and volume rendering (VR). According to the need, the appropriate reconstruction method and reconstruction parameters were selected. The heart and great vessels were displayed in different directions, and the lesions were observed from different angles. The width of 5 levels, including the ascending aorta, precoarctation aorta, site of coarctation, postcoarctation, and descending aorta at the level of the diaphragm, was measured (Figure 1). The values were normalized to a Z score based on the square root of body surface area.

## Surgical method

The infants were placed in the supine position, and preoperative blood pressure of the upper and lower extremities was measured by invasive arterial blood pressure monitoring. Then, the patient was placed in a 90-degree lying position on the right side, and the left surgical field was routinely disinfected and draped. The skin, subcutaneous tissue, and back muscles were sequentially incised, and thoracotomy was performed through the fourth intercostal space. The posterior pleura and adventitia of the descending aorta were longitudinally incised. The lower segment of the aortic arch, branch vessels, and the proximal end of the descending aorta were carefully identified and dissociated, and the position of the coarctation was found. In the case of discrete CoA, simple end-to-end anastomosis was performed by blocking the superior and inferior normal arteries and the corresponding branch arteries with blocking forceps and continuous anastomosis at both ends of the aorta. In cases of combined aortic arch hypoplasia, resection and extended end-to-end anastomosis were performed. The aortic arch was controlled near the left carotid artery. Distal blocking forceps were placed distal to the constricted area. After complete excision of all constricted and conduit tissue, the inferior surface of the transverse aortic arch was dissected until a partial and end-to-end anastomosis between the cephalic brachial artery and the left common carotid artery was made. After completion of the anastomosis, blood pressure was measured by invasive arterial blood pressure monitoring in the upper and lower extremities. Prior to closure a thoracic drainage tube was inserted and the wound was closed in layers. The infants were transferred to the ICU after surgery.

## Data collection

The relevant data needed in this study were collected by searching medical electronic record systems. The aortic coarctation data of all patients measured by preoperative CTA were collected, and patients were divided into the mild CoA group and the severe CoA group according to the degree of coarctation. The sex, prenatal diagnosis, gestational age, age at operation, presurgical weight, surgical duration, aortic cross-clamp time, mechanical ventilation duration, length of ICU stay, and other clinical data of the two groups of patients were collected. All patients were followed up regularly after discharge. Patients typically received initial TTE and further CTA if re-CoA occurred. The relevant clinical information in this cohort was collected for research purposes, and data collection was performed in a strictly confidential manner.

The outcomes evaluated in this study were divided into three categories. (1) We compared the differences in prognosis-related data, such as mechanical ventilation duration and length of ICU stay, between the two groups. (2) We assessed the relationship among sex, gestational age, age at operation, presurgical weight, severity of CoA, aortic cross-clamp time, duration of mechanical ventilation and postoperative ICU length of stay. (3) We assessed the relationship between the degree of CoA and z scores at different levels of the aorta.

## Statistical analysis

Data for this study were analyzed using IBM SPSS Statistics version 23. Qualitative variables were expressed as frequencies and percentage values (%), and quantitative variables were defined as the mean ± standard deviation. This study used Q-Q plots to analyze whether variables were normally distributed. Due to the small sample size of this study, Fisher's exact probability method was used instead of *χ*^2^ for analysis. Multiple linear regression models were used to assess independent associations between postoperative ICU stay and relevant demographic and clinical factors. Spearman correlation analysis was used to evaluate the relationship between the degree of coarctation of the aorta and the Z score at different levels of the aorta. A *P* value less than 0.05 was considered statistically significant.

## Results

A total of 27 infant patients with simple CoA were included in this study. The preoperative data of the patients and the relevant data at the time of surgery are shown in Table 1. There were no significant differences in sex, gestational age, age at diagnosis, preoperative pulmonary artery pressure, left ventricular ejection fraction, aortic cross-clamp time or surgical duration between the two groups.

**Table 1:** Comparison of preoperative general clinical data between the two groups.

	Mild group (*n* = 13)	Severe group (*n* = 14)	*P* value
Male gender, *n* (%)	8 (61.5)	7 (50.0)	0.704
Prenatally diagnosed, *n* (%)	2 (15.4)	5 (35.7)	0.385
Gestational age, *w*	36.5 ± 1.8	36.3 ± 2.0	0.823
Age at diagnosis, *d*	13 ± 5.1	9 ± 4.5	0.712
Age at operation, *d*	18.4 ± 8.1	16.3 ± 7.1	0.483
Presurgical weight, kg	3.2 ± 0.6	3.3 ± 0.8	0.851
Pulmonary artery pressure, mmHg	34.2 ± 5.1	38.7 ± 7.2	0.069
Left ventricular ejection fraction	64.5 ± 7.6	60.7 ± 6.8	0.182
Preoperative prostaglandin, *n* (%)	2 (15.4)	4 (28.6)	0.648
Preoperative mechanical ventilation, *n* (%)	1 (7.7)	3 (21.4)	0.596
Z score of aorta			
Ascending aorta	2.03 ± 0.41	2.39 ± 0.37	0.015
Pre-coarctation	1.67 ± 0.38	1.81 ± 0.44	0.353
Post-coarctation	1.73 ± 0.33	2.09 ± 0.28	0.021
Operation data			
Aortic cross-clamp time, min	15.2 ± 2.5	14.4 ± 4.3	0.557
Surgical duration, min	97.9 ± 27.1	106.9 ± 17.1	0.398

Table 2 shows no significant difference in postoperative blood transfusion, postoperative pneumonia, peritoneal dialysis, and mortality between the two groups. The analysis showed that the duration of mechanical ventilation and the length of ICU stay of the mild CoA group were significantly shorter than those in the severe CoA group. The comparison of follow-up data revealed no significant difference in follow-up time or the incidence of postoperative re-CoA between the two groups.

**Table 2:** Comparison of postoperative clinical results between the two groups.

	Mild group (*n* = 13)	Severe group (*n* = 14)	*P* value
Postoperative blood transfusion, *n* (%)	3 (23.1)	5 (35.7)	0.678
Postoperative pneumonia, *n* (%)	5 (38.5)	8 (57.1)	0.449
Feeding intolerance, *n* (%)	2 (15.4)	4 (28.6)	0.648
Peritoneal dialysis, *n* (%)	0 (0)	3 (21.4)	0.222
Duration of mechanical ventilation, *d*	3.4 ± 1.6	5.1 ± 1.8	0.002
Total LOS in CICU, *d*	5.7 ± 2.1	7.8 ± 2.5	0.001
Mortality, *n* (%)	0 (0)	2 (14.3)	0.481
Follow-up, *m*	8.3 ± 2.5	7.9 ± 3.4	0.559
Recoarctation, *n* (%)	1 (0)	3 (21.4)	0.596

^a^ LOS, length of stay.

Table 3 shows the results of the multiple linear regression analysis. CoA severity was a significant predictor of prolonged postoperative ICU stay. In addition, gestational age and age at operation were also identified as risk factors for a prolonged ICU stay. We analyzed the correlation between the degree of aortic coarctation and the Z scores of the different aortic sites. The results showed that the degree of aortic coarctation was correlated with the Z scores of the ascending aorta and postcoarctation aorta. (Table 4).

**Table 3:** Multiple linear regression analysis of the different predictors of total length of ICU stay.

	OR (95% CI)	*P* value
Male gender	0.59 (−0.28, 1.45)	0.171
Gestational age	−0.36 (−0.69, −0.04)	0.032
Age at operation	−0.08 (−0.16, −0.01)	0.047
Presurgical weight	0.08 (−0.57, 0.74)	0.793
Severity of CoA	1.71 (1.19, 2.73)	0.002
Aortic cross-clamp time	−0.15 (−0.23, 0.02)	0.090
Duration of mechanical ventilation	0.42 (0.03, 0.87)	0.062

**Table 4:** Correlation of the z score of aorta and degree of coarctation.

	R (correlation coefficient)	*P* value
Ascending aorta	0.493	0.009
Pre-coarctation aorta	0.517	0.225
Post-coarctation aorta	0.198	0.018

## Discussion

This study was a retrospective analysis of the effect of the degree of aortic coarctation on the prognosis of infants undergoing surgical correction of simple CoA. The results showed that the duration of postoperative ICU stay was significantly shorter in infants with mild CoA than in infants with severe CoA. Multiple linear regression analysis showed that the severity of aortic coarctation was a significant predictor of postoperative ICU stay. In addition, gestational age and operation age were significant predictors of postoperative ICU stay. Correlation analysis indicated that the degree of aortic coarctation significantly correlated with the Z scores of the ascending aorta and the postcoarctation aorta.

In recent years, significant progress has been made in studying factors influencing the short-term and long-term outcomes of patients with coarctation of the aorta. The anatomical structure of the patient's aorta, nutritional status, surgical methods, and surgical approaches influence surgical prognosis (9–11). This study further identified risk factors associated with prolonged ICU stay in infants with simple CoA. Among them, the most important finding was that the degree of aortic coarctation was an important risk factor for a prolonged ICU stay. Previous studies on the outcome of aortic surgery have rarely associated the severity of aortic coarctation with poor surgical outcomes. Preeti et al. showed that aortic arch size might play a positive role in the long-term outcome, but whether it was a predictor of recoarctation or long-term outcome was unclear (12). The results of our study were positive, as the length of ICU stay in the severe group was significantly longer than that in the mild group. Previous studies have shown that the reason for the poor prognosis of aortic arch surgery might be related to altered arterial responsiveness, changes in vascular resistance, and changes in blood flow distribution caused by aortic coarctation, and these effects are exacerbated by the degree of aortic coarctation (13–15). In previous studies on the predictive effect of preoperative aortic size on the occurrence of postoperative aortic re-CoA, different scholars had different opinions. Dongngan et al. showed that a smaller aortic size measured by preoperative TTE was associated with postoperative re-CoA (16). However, Kaushal et al. suggested that reconstriction was not associated with arch hypoplasia and coarctation size (17). In our results, the degree of aortic coarctation found during the follow-up of infants with CoA after surgery did not independently predict postoperative aortic recoarctation.

In recent years, with the rapid development of auxiliary examination technology, numerous auxiliary means are available for diagnosing CHD, including echocardiography, CT, and magnetic resonance imaging (MRI). Currently, TTE remains the most commonly used method to diagnose CHD. However, TTE imaging has significant limitations in assessing aortic lesions due to its poor quality and occasional inaccuracies in assessing aortic branching vessels (18). CTA, as a developed imaging technique, has high spatial resolution and a wide field of view, which is helpful to comprehensively describe the CoA and help surgeons completely understand the condition of aortic coarctation (19). The predictive effect of aortic coarctation on surgical prognosis further confirmed the necessity of preoperative CTA examination. In addition, we also found that the degree of aortic coarctation significantly correlated with the Z scores of the ascending aorta and the postcoarctation aorta, which was similar to that noted in previous studies (20). These findings were extended further to infants with simple CoA. This phenomenon might be related to the aortic stress difference and hemodynamic factors (21, 22). At present, no definitive conclusion has been reached to explain this phenomenon. The importance of examining aortic coarctation by CTA should be noted when perfecting preoperative examination.

The number of young, low-weight infantile patients undergoing surgical repair of CoA has increased significantly in recent years. Rinske et al. suggested that age was one of the predictors of longer ICU stay after surgery. Our findings also found that age at aortic surgery was an independent risk factor associated with a longer ICU stay. The effect of age at the time of surgery on postoperative ICU stay might be explained by various factors, such as younger age, incomplete development, and poor resistance (23, 24). Considering that the difference in gestational age might still be large if the infants were differentiated according to whether they were preterm, this study directly used gestational age as a research factor, and the analysis showed that infants with younger gestational age had a higher risk of poor prognosis after surgery. The results were similar to those noted in previous studies (25, 26). Therefore, the timing of surgery is very important for the surgical outcome, but consensus on the optimal timing is lacking. Further research is needed to determine the most appropriate age for such surgery.

## Limitations

Some limitations in this study should be noted. First, due to the low incidence of simple coarctation of the aorta, the sample size that could be included in this study was limited. The small sample size of our study somewhat limited our ability to determine the association between the degree of aortic coarctation as a predictor and the length of postoperative ICU stay. However, we still believe that our results have some clinical implications. Second, this study was a single district retrospective study, and relevant data were not collected from different areas. Moreover, our postoperative follow-up time was limited; thus, the impact of postoperative recoarctation was potentially not accurately assessed. Further prospective studies are needed in the future to more comprehensively and rationally assess the association between the degree of aortic coarctation and surgical prognosis.

## Conclusion

The degree of aortic coarctation is an important predictor of poor surgical outcomes in infants with simple CoA. The degree of aortic coarctation significantly correlates with the Z scores of the ascending aorta and the postcoarctation aorta. Therefore, CTA should be routinely performed before aortic surgery to assess the degree of coarctation and better identify risk factors.

## Data Availability

The original contributions presented in the study are included in the article/Supplementary Material, further inquiries can be directed to the corresponding author/s.
